# Th17/Treg Imbalance and Atherosclerosis

**DOI:** 10.1155/2020/8821029

**Published:** 2020-10-31

**Authors:** Xin He, Bo Liang, Ning Gu

**Affiliations:** ^1^Nanjing University of Chinese Medicine, Nanjing, China; ^2^Nanjing Hospital of Chinese Medicine Affiliated to Nanjing University of Chinese Medicine, Nanjing, China

## Abstract

Atherosclerosis is nowadays recognized as a chronic inflammatory disease of large arteries. In recent years, cellular and molecular biology studies on atherosclerosis confirmed that the occurrence and development are related to inflammation and autoimmunity. A variety of immune cells, cytokines, and transcription factors are involved in this process. Current studies found that T helper cell 17, regulatory T cells, and their cytokines play an important role in the development of atherosclerosis and vulnerable plaque rupture. Here, we provide a review of the up-to-date applications of T helper cell 17, regulatory T cells, cytokines, and their balance in the prognosis and therapy of atherosclerosis.

## 1. Introduction

Atherosclerosis, a pathological condition that underlies several important adverse vascular events, including coronary artery disease (CAD), stroke, and peripheral arterial disease, is responsible for most of the cardiovascular morbidity and mortality in the world today [1]. CAD is the further development of atherosclerosis, and its main pathological process is the activation of inflammatory reactions and the coagulation system [2]. A variety of inflammatory cells and cytokines contribute to the thinning of the fibrous cap and enlargement of the lipid core, thus promoting the formation and rupture of vulnerable plaques. Pathogenesis of atherosclerosis includes lipid infiltration, damage response, monocular macrophage invasion, and inflammation response [3]. Currently, atherosclerosis is considered to be a vascular wall chronic inflammatory disease and involves cellular immune responses. T cells predominantly populate atherosclerotic lesions with an enrichment in the fibrous cap [4, 5]. In terms of adaptive immune response, CD4^+^ T helper cell 1 (Th1) and T helper cell 2 (Th2) are regarded as important factors regulating immune balance. Suppression of Th1 function or enhancement of Th2 function has been proven to reduce atherogenesis in *apolipoprotein E (ApoE)^−/−^* or l*ow-density lipoprotein receptor (Ldlr)^−/−^* mice [6–8]. In a mouse model resistant to atherosclerosis, an increase of Th2 protects against early fatty streak development [9]. However, increased evidences indicated that T helper cell 17 (Th17) and regulatory T cells (Treg) are highly involved in atherogenesis [10]. T cells present during all stages of the disease are essential to the development of atherosclerotic plaque. Among them, Th17-mediated proinflammatory responses aggravate atherosclerosis while Treg play a key atheroprotective role by limiting inflammation and counterbalancing plaque formation [11]. Recently, studies found that the Th17/Treg balance could control inflammation and may play an important role in the plaque stability. Therefore, the balance between Th17 and Treg may be important for the development and prevention of atherosclerosis. Here, we critically review the related cytokines, transcription factor, Th17, Treg, and their imbalance in atherosclerosis in order to contribute to the knowledge concerning atherosclerosis pathogenesis.

## 2. Cytokines

Cytokines are small molecular polypeptides or glycoproteins synthesized and secreted by a variety of tissue cells (mainly immune cells). Cytokines can mediate the interaction between cells and have a variety of biological functions. Emerging evidences show that the interleukin (IL) and transforming growth factor-*β* (TGF-*β*) families, two kinds of cytokines, play an important role in the occurrence and development of atherosclerosis [12, 13].

### 2.1. IL-10

IL-10 is an inhibitory cytokine produced by activated lymphocytes and monocytes, thought to be protective against the development and progression of atherosclerosis. IL-10 suppresses antigen presenting capacity, dendritic cell activity, and T cell proliferation, as well as negatively regulates proinflammatory cytokine production [14, 15]. It is an anti-inflammatory cytokine, deficiency of which increases atherosclerosis in atherosclerosisprone mice [16]. Mallat et al. showed that IL-10 deficiency in C57BL/6 mice fed an atherogenic cholate-containing diet promotes early atherosclerotic lesion formation, characterized by increased infiltration of inflammatory cells, particularly activated T cells, and by increased production of proinflammatory cytokines [17]. Data suggests that a higher level of IL-10 at the time of an acute coronary syndrome event is protective against risk for future cardiovascular events [18, 19]. Animal data suggest that IL-10 prevents atherosclerotic plaque development, improves plaque stability, and promotes lesion size reduction [8, 20, 21]. Studies evidenced that IL-10 can inhibit minimally oxidized low-density lipoprotein-induced monocyte-endothelium interaction [16] and increase actin filament rearrangement in macrophages, and induces the uptake of high-density lipoprotein and low-density lipoprotein by fluid-phase endocytosis [22], thus inhibiting atherosclerotic lesion formation in mice fed an atherosclerotic diet. Systemic or local overexpression of IL-10 by adenoviral gene transfer in a model of collar-induced atherosclerosis in *Ldlr^−/−^* mice was found highly efficacious in preventing atherosclerosis [23], and overexpression of IL-10 by activated T lymphocytes reduced atherosclerosis in *Ldlr^−/−^* mice [8]. More recently, using a model of chimeric *Ldlr^−/−^* mice in which bone marrow cells were deficient for IL-10, we provided evidence that leukocyte-derived IL-10 is instrumental in the prevention of atherosclerotic lesion development and in the modulation of cellular and collagen plaque composition, at least in part, through a systemic immune response modulation [7].

### 2.2. IL-17

In immune, endothelial, and stromal cells, IL-17 induces the secretion of the proinflammatory cytokine IL-6, granulocyte colony-stimulating factor, and granulocyte-macrophage colony-stimulating factor, as well as chemokines, all of which can be proatherogenic [24]. By contrast, IL-6 and TGF-*β* induce a subtype of Th17 cells that produces IL-10 concomitantly with IL-17, and IL-10 can be atheroprotective [16, 25, 26]. ApoE is a protein in plasma and plays a vital role in lipid metabolism. It had been confirmed that lack of ApoE resulted in accumulation in plasma of cholesterol-rich remnants and thus induced atherosclerosis. So, the *ApoE^−/−^* mouse is widely used in the research for atherosclerosis as it can manifest pathological features of human atherosclerosis [27, 28]. Experimental studies in *ApoE^−/−^* mice on the role of IL-17 yielded discrepant results, with some studies suggesting that IL-17A is proatherogenic [40] and others atheroprotective [29] and a further study suggesting that IL-17 has no effect on atherosclerosis [30]. Some studies in mouse models of atherosclerosis demonstrated that IL-17 can promote plaque stability by increasing the production of type I collagen by vascular smooth muscle cells (VSMC) [49, 61]. Moreover, IL-17 signaling activates various downstream pathways, which include nuclear factor kappaB (NF-*κ*B) and mitogen-activated protein kinases to induce various mediators with relevance to atherosclerosis. The NF-*κ*B transcription factor was discovered 30 years ago and has since emerged as the master regulator of inflammation and immune homeostasis. It achieves this status by means of the large number of important pro- and anti-inflammatory factors under its transcriptional control. NF-*κ*B has a central role in inflammatory diseases such as atherosclerosis. NF-*κ*B is an evolutionarily conserved transcription factor that provides a means to achieve inducible, specific, and regulated immune responses [31]. The proinflammatory nature of the transcriptional targets of NF-*κ*B and their inherent potential for damage to host tissue necessitates tight control of NF-*κ*B activation and transcriptional activity. The consequences of uncontrolled, inappropriate, or dysregulated inflammation are manifested in a range of diseases including atherosclerosis. Many studies identified additional mechanisms, mostly involving posttranslational modification of the NF-*κ*B subunits, that regulate NF-*κ*B-mediated transcriptional responses. An array of posttranslational modifications is identified including phosphorylation, ubiquitination, acetylation, glycosylation, and nitrosylation, all of which directly affect NF-*κ*B transcriptional activity [32]. A study showed that IL-17 can promote the expression of vascular cell adhesion molecule-1 in aortic VSMC by inducing activation of NF-*κ*B, which is important for the development of atherosclerosis. Therefore, these signaling pathways might be therapeutic targets for treatment of IL-17-mediated inflammation [33]. IL-17 alone often stimulates a weak response, but it may synergize with different cytokines like tumor necrosis factor-*α*, interferon-*γ*, granulocyte-macrophage colony stimulating factor (GM-CSF), IL-1*β*, and IL-22 to increased production of inflammatory mediators such as IL-6 and IL-8, leading to increased and prolonged proinflammatory response [34]. On the other hand, the antiatherogenic impact of IL-17 may be referring to its inhibitory action on vascular cell adhesion molecule-1 expression and inflammatory adhesion molecules on fibroblasts and VSMC [35]. Besides, elevated systemic levels of the acute phase C-reactive protein are predictors of future cardiovascular events. The results, confirmed in primary human hepatocytes and coronary artery smooth muscle cells, demonstrate for the first time that IL-17 is a potent inducer of C-reactive protein expression via p38 mitogen-activated protein kinase and extracellular regulated protein kinase 1/2-dependent NF-*κ*B and CCAAT/enhancer binding protein *β* activation and suggest that IL-17 may mediate chronic inflammation, atherosclerosis, and thrombosis [36]. In some clinical studies, it is shown that plasma IL-17 levels and the number of peripheral Th17 cells are increased in patients with unstable angina or acute myocardial infarction compared with patients with stable angina and healthy individuals [37, 38]. Erbel et al. administered *in vivo* IL-17-blocking antibody in *ApoE^−/−^* mice and found that functional blockade of IL-17 reduced atherosclerotic lesion improvement and lowered plaque vulnerability, cellular infiltration, and tissue activation [39]. They concluded that IL-17 plays a pivotal role in atherogenesis.

Retinoic-related orphan receptor- (ROR-) *γ*t is an isoform of ROR-*γ* that belongs to the retinoid acid-related orphan receptor subgroup; it is best known as the regulator of Th17 cells and more broadly the transcription factor controlling IL-17 production in other cells [40, 41]. ROR-*γ*t plays a crucial role in the induction of autoimmune tissue injuries and inflammation [42]. ROR-*γ*t cooperates with other transcriptional factors, including signal transducer and activator of transcription 3 and runt-related transcription factor 1, to induce IL-17 expression; the transcription factor basic leucine zipper transcription factor controls the differentiation Th17 cells by regulating the expression of ROR-*γ*t [43, 44]. Ubiquitinylation and deubiquitinylation is an inverse process that can regulate protein stability dynamically. It is found that TGF-*β* plus IL-6 which are important signals for Th17 differentiation could enhance the deubiquitinylation mediated by Ubiquitin-Specific Peptidase 4, which could promote ROR-*γ*t function, suggesting that Ubiquitin-Specific Peptidase 4 may play a role in Th17 development [45]. Transcription factors are also involved in the regulation of ROR-*γ*t. A study established that Forkhead Box P3 (Foxp3) can inhibit ROR-*γ*t transcriptional activity during Th17 differentiation [46].

Recently, a study demonstrated that loss of the immune-regulatory factor tripartite motif containing 21 (Trim21) influences the atheromatous process. Trim21, as a ubiquitin E3 ligase, is the effector ligase in the ubiquitination cascade; the main role has been implicated in immune processes. The research showed that Trim21-deficient bone marrow transplanted into *Ldlr^−/−^* mice fed a hypercholesterolemic diet would develop larger atherosclerotic plaques with significantly higher collagen content. *Ldlr^−/−^* mice are one of the most widely used genetically engineered animals in the field of atherosclerosis. Compared with *ApoE^−/−^* mice, the lipoprotein profile of *Ldlr^−/−^* mice is closer to humans, which is helpful to infer the relationship between lipoprotein changes and human atherosclerosis and hyperlipidemia [47]. The data showed that TRIM21 deficiency promotes IL-17 expression, smooth muscle cell levels increased, and protein expression levels of interferon-*γ* and matrix metalloproteinases (MMPs) decreased in mice. The result indicated that Trim21 affects atherosclerosis by regulating the Th17 response, promoting plaque fibrosis and stability [48].

Furthermore, the detrimental effects of a high-salt diet on human health have received much attention in the past few years. It has been well established that high dietary salt intake is related to cardiovascular diseases; most studies discussing the mechanism for the detrimental effect of high salt demonstrated a pivotal involvement of pathogenic Th17 cells. In humans, GM-CSF expression was shown to be inhibited by the IL-23/ROR-*γ*t/Th17 axis [49]. A study indicated that high sodium concentrations increased the differentiation of murine and human Th17 cells and induced a highly pathogenic phenotype, characterized by an increased expression of the surface receptors IL-23 receptor and chemokine receptor C-C motif chemokine receptor 6 and the upregulation of GM-CSF, IL-2, and tumor necrosis factor-*α*. This plays an important role in the occurrence and development of atherosclerosis [50]. In addition to the increased induction of proinflammatory Th17 cells, excess dietary sodium intake can impact autoimmunity by reducing the number of Treg and inhibiting the function of Treg.

### 2.3. IL-35

IL-35, a novel functional cytokine of Treg comprised of the IL-12p35 subunit and the other subunit Epstein-Barr virus-induced gene 3, regulates the activity of CD4^+^ T cells and macrophages, thereby playing a critical role in atherosclerosis [51]. In a study, researchers examined the expression of IL-35 during early atherosclerosis and found that IL-35 blocks lysophosphatidylcholine-induced mitochondrial reactive oxygen species, which are required for the induction of site-specific histone 3 lysine 14 acetylation, increased binding of proinflammatory transcription factor activator protein-1 in the promoter of intercellular adhesion molecule-1, and induction of intercellular adhesion molecule-1 transcription in human aortic endothelial cells. It indicated that IL-35 is induced during atherosclerosis development and inhibits mitochondrial reactive oxygen species-histone 3 lysine 14 acetylation-activator protein-1-mediated endothelial cell activation [52]. Recently, Huang et al. found that in *ApoE^−/−^* mice, IL-12p35 deficiency reduces the level of IL-35, inhibits the generation and function of Treg, exacerbates Th17/Treg imbalance, promotes atherosclerosis, but stabilizes the plaque [51]. In animal studies, treatment with recombinant human IL-35 led to an increase in both circulating and local Treg levels and a reduction in the plaque size in *ApoE^−/−^* mice, suggesting that IL-35 attenuates atherosclerosis via upregulating Treg immune response [53]. Taken together, these results indicated that IL-35 exerts an antiatherosclerotic effect and facilitates stability of the vulnerable plaques by increasing Treg levels.

### 2.4. TGF-*β*

TGF-*β* is a potent anti-inflammatory, immune-suppressive, and profibrotic cytokine, with major effects on the biology of VSMC [54]. The anti-inflammatory and profibrotic properties of TGF-*β* highly suggest the potential antiatherogenic role for this cytokine. Recent advances in the study of atherosclerosis point to an important role of TGF-*β* signaling in the protection against excessive plaque inflammation, loss of collagen content, and induction of regulatory immunity [54]. Specific abrogation of TGF-*β* receptor 2 signaling in T cells aggravates atherosclerosis and causes an inflammatory and destabilized plaque phenotype [55]. Overexpressing TGF-*β* in hearts of *ApoE^−/−^*mice decreases lesion size, reduces T cell infiltration, and increases collagen production in the plaques, demonstrating the critical role of TGF-*β* for VSMC matrix production and plaque stability in atherosclerosis [56]. TGF-*β*-triggered signals are transduced by proteins belonging to the SMAD family [57]. Immunohistochemistry and reverse transcription-polymerase chain reaction analysis of human plaques reveal SMAD family member 2, SMAD family member 3, and SMAD family member 4 expression in macrophages of fibrofatty lesions and in VSMC of fibrous caps [58]. Mallat et al. also detected phosphorylated SMAD family member 2 in the aortic sinus of *ApoE^−/−^* mice, indicative of TGF-*β* activity in atherosclerotic lesions [59]. SMAD family member 7 (Smad7) is viewed as a major inhibitory regulator of TGF-*β* signaling. Bone marrow from mice with a T cell-specific deletion of Smad7, a potent inhibitor of TGF-*β* signaling, was transplanted into hypercholesterolemic *Ldlr^−/−^* mice. Smad7-deficient mice had significantly larger atherosclerotic lesions that contained large collagen-rich caps, consistent with a more stable phenotype [60]. Taken together, these results show that abrogation of TGF-*β* signaling in T cells increases atherosclerosis and suggest that TGF-*β* reduces atherosclerosis by dampening T cell activation [55, 61]. The important role of TGF-*β* signaling in atherosclerosis suggests that regulatory pathways in adaptive immunity are essential in modulation of the development and progression of atherosclerosis.

## 3. Foxp3

Foxp3 is a marker of human Treg, which is critical for anti-inflammatory responses and for maintaining immune tolerance mainly by regulating the secretion of the anti-inflammatory cytokine IL-10 [62]. TGF-*β* plus T cell receptor stimulation triggers naive CD4^+^ CD25^−^ Foxp3^−^T cells to differentiate into Foxp3^+^Tregs, via a SMAD-dependent pathway in mice [63, 64]. It has been reported that a number of factors can influence the expression of Foxp3 including conserved noncoding DNA sequence at the Foxp3 gene locus and transcription factors such as NF-*κ*B, nuclear factor of activated T cells, SMAD family member 3, signal transducers, and activators of transcription 5 [65]. Using transgenic mice with increased or decreased NF-*κ*B activity, Long et al. showed that the T cell receptor-induced NF-*κ*B pathway upregulates Foxp3 expression [66]. A clinical study showed that CD31 signal transduction mediated by recruitment and activation of tyrosine phosphatases, resulting in attenuated Foxp3 expression leads to impaired secretion of inhibitory cytokines and subsequent suppressor function of Treg cells [11]. According to a study, Foxp3 can be regulated by various posttranscriptional modifications including ubiquitinylation, acetylation, and phosphorylation, which can influence both its stability and function [65]. An Foxp3 E3 ligase STIP1 homology and u-box containing protein 1 (STUB1) was recently identified by Chen et al., and it targets Foxp3 to degradation by promoting K48-linked polyubiquitinylation of Foxp3 in a heat shock protein 70-dependent manner. Based on the role STUB1 plays in regulating the stability of Foxp3, targeting STUB1 may be beneficial in the inflammation conditions of atherosclerosis [67]. Li et al. found that TGF-*β* could increase the acetylated level of Foxp3 and promote the recruitment of Foxp3 to IL-2 promoter. Inversely, IL-6 treatment reverses this effect, suggesting that the inflammatory signals downregulate Treg function partially through regulating Foxp3 acetylation. Further, histone deacetylation inhibitor treatment abolished the decreased acetylation of Foxp3 by TGF-*β* and IL-6, suggesting that TGF-*β* and IL-6 may upregulate histone deacetylation to deacetylate Foxp3 [68].

## 4. Immune Cells

### 4.1. Th17

In the past decade, increasing attention has been focused on a subset of CD4^+^ T cells, commonly known as Th17 cells. Th17, the third subpopulation of Th cells, plays critical roles in the development of autoimmunity and allergic reactions and recently has been thought to be key regulators of inflammation and thus may potentially contribute to the immunopathogenesis of atherosclerosis [69, 70]. Th17 cells are characterized by expression of the Th17-defining transcription factor nuclear receptor retinoic-related orphan receptor- (ROR-) *γ*t. Th17 cells are activated by IL-23, and IL-17 is their main secreted cytokine [24].

### 4.2. Treg

Naturally arising Treg cells, most of which are produced by the normal thymus as a functionally mature T cell subpopulation, play key roles in the maintenance of immunologic self-tolerance and negative control of a variety of physiological and pathological immune responses. Treg can be divided into natural Treg and inducible Treg and plays a significantly protective role in atherosclerosis by limiting inflammation and stabilizing plaque. Treg cells exert their atheroprotective properties by secreting IL-10 and TGF-*β* and by suppressing the proliferation of proinflammatory effector T cells [71]. In mice, Treg cells protect against atherosclerosis [72]. Similarly, clinical data suggest a strong inverse relationship between Treg cells and atherosclerosis, whereby Treg cell numbers and IL-10, a cytokine secreted by Treg cells, are lower in patients with myocardial infarction than in patients with stable angina or individuals without coronary artery disease [73, 74]. Studies showed that the generation of Treg cells induced by immunity can reduce atherosclerosis in mice [75–77].

### 4.3. Th17/Treg Imbalance

The Th17/Treg is a newly balanced pair which plays an important role in the development of atherosclerosis and plague rupture [78]. Various signals, factors, epigenetic modifications, metabolic pathways, and microbiota are shown to regulate the plasticity between Tregs and Th17 cells [65]. A clinical study showed that the Th17/Treg imbalance might act synergistically with microinflammation on immune-mediated atherosclerosis and contribute to the high incidence of adverse cardiovascular events [79]. Compared with healthy people, the number of Th17 in the peripheral blood of CAD patients and IL-17, IL-6, IL-23, and ROR-*γ*t levels increased significantly; the number of Treg, IL-10, TGF-*β*, and Foxp3 and the ratio of Treg and Th17 decreased significantly. The results showed that patients with CAD had significant Th17/Treg imbalance, suggesting the potential role of Th17/Treg imbalance in plaque instability and CAD episodes [37]. Numerous animal studies have proven that reversing the imbalance of Th17/Treg significantly attenuated atherosclerosis by drugs. Peroxisome proliferator-activated receptors could regulate Th17 and Treg plasticity by enhancing Foxp3 expression and decreasing ROR-*γ*t and IL-17A expression. Pioglitazone, an agonist of peroxisome proliferator-activated receptors, could stabilize atherosclerotic plaque by inducing protein phosphorylation, decreasing IL-17, and increasing Foxp3 cells, improving Th17/Treg balance in the spleen of *ApoE^−/−^* mice [80]. The Angong Niuhuang pill, a Chinese traditional medicine, has been proven to protect atherosclerotic *ApoE^−/−^* mice by regulating Th17/Treg balance, inhibiting chronic inflammation, reducing plaque collagen fibers, and reducing inflammatory cell infiltration, which are probably related to regulating ROR-*γ*t and Foxp3 expression [81]. In addition, a recent clinical study shows that after statin treatment, the number of Th17 and accumulation of IL-17, IL-6, and IL-23 decreased and the number of Treg and accumulation of IL-10 and TGF-*β* increased in the peripheral blood in CAD patients. It indicated that statin therapy could ameliorate the Th17 and Treg functional imbalance in patients with CAD. Thus, Th17/Treg imbalance plays an important role in the development of atherosclerosis and plaque instability. Drug therapy can reverse the Th17/Treg imbalance, delaying the progress of atherosclerosis and stabilizing plaque.

## 5. Conclusion

Atherosclerosis is the main cause of most cardiovascular and cerebrovascular diseases. The activation of immunity is closely related to atherosclerosis; at the same time, the imbalance of regulatory and pathogenic immunity may promote the development of atherosclerosis. The balance between pro- and anti-inflammatory cytokines has emerged as a major determinant of atherosclerosis. Therefore, exploring the Th17/Treg imbalance could provide a new idea and target for the treatment of atherosclerosis.

## Data Availability

The data used to support the findings of this study are included within the article.
